# T-cell receptor signaling in Schimke immuno-osseous dysplasia is SMARCAL1-independent

**DOI:** 10.3389/fimmu.2022.979722

**Published:** 2022-10-18

**Authors:** Ana V. Marin, Anaïs Jiménez-Reinoso, Marina S. Mazariegos, Elena Román-Ortiz, José R. Regueiro

**Affiliations:** ^1^ Department of Immunology, Ophthalmology and Ear, Nose and Throat (ENT), Complutense University School of Medicine and 12 de Octubre Health Research Institute (imas12), Madrid, Spain; ^2^ Pediatric Nephrology, Hospital Universitari i Politècnic la Fe, Valencia, Spain

**Keywords:** Schimke, SIOD, SMARCAL1, immunosuppression, T-cell, lymphopenia, kidney failure

## Abstract

Schimke immuno-osseous dysplasia (SIOD) caused by mutations in *SMARCAL1* is an ultra-rare disease characterized by specific facial features, skeletal dysplasia, and steroid-resistant nephrotic syndrome, which often leads to kidney failure and requires transplantation. Cellular (T-cell) deficiency, lymphopenia, and infections have been frequently reported, but whether they are due to T-cell-intrinsic defects in T-cell receptor (TCR) signaling associated with SMARCAL1 deficiency or to T-cell-extrinsic effects such as the impaired proliferation of hematopoietic precursors or T-cell-specific immunosuppression after renal transplantation remains unknown. We have explored the effects of SMARCAL1 deficiency on T-cell receptor signaling in primary and immortalized T cells from a 9-year-old SIOD patient under immunosuppression treatment when compared to healthy donors. Immortalized T cells recapitulated the SMARCAL1 deficiency of the patient, as judged by their impaired response to gamma irradiation. The results indicated that TCR-mediated signaling was normal in SIOD-derived immortalized T cells but strongly impaired in the primary T cells of the patient, although rescued with TCR-independent stimuli such as PMA + ionomycin, suggesting that SIOD-associated T-cell signaling is not intrinsically defective but rather the result of the impaired proliferation of hematopoietic precursors or of T-cell-specific immunosuppression. The lack of early thymic emigrants in our patients may support the former hypothesis.

## Introduction

Schimke immuno-osseous dysplasia (SIOD) is a multisystem disorder that is inherited in an autosomal recessive pattern and characterized by the combination of specific facial features, skeletal dysplasia, steroid-resistant nephrotic syndrome, and defective cellular immunity with episodic severe lymphopenia. Less than 100 cases have been reported and its prevalence is 1 in 1–3 million new births, so Orphanet considers it an ultra-rare disease (OMIM: 242900). Early-onset affected patients show severe symptoms and die at around 10 years of age due to strokes, severe opportunistic infections, bone marrow failure, kidney failure, cardiovascular issues, and other complications (1).

SIOD is the consequence of biallelic mutations in *SMARCAL1* (SWI/SNF-related Matrix-associated Actin-dependent Regulator of Chromatin, subfamily A-Like 1), also known as HARP or HepA-Related Protein. SMARCAL1 is a member of the Sucrose Non-Fermenting 2 (SNF2) family of ATP-dependent chromatin remodeling enzymes, highly conserved in evolution. It is expressed in the nucleus and has a role in the maintenance of genome stability and reactivation of stalled DNA replication forks (2). The N-terminal domain of SMARCAL1 contains a replication protein A (RPA)-binding region followed by two tandem HARP domains. SMARCAL1 catalyzes replication fork remodeling when it binds to RPA to maintain genome stability activity (3) and has annealing ATP-dependent helicase activity relying on the HARP domains (4). The C-terminal domains of SMARCAL1 contain the SNF2 ATPase region, composed of a region shared by the SNF2 family, the Helicase ATP-binding region, and a Helicase C-terminal region, both necessary to bind and hydrolyze ATP and transduce the resulting energy for chromatin remodeling (5). *SMARCAL1* mutations accumulate in the Helicase ATP-binding region, but mutations along the entire gene have been reported (6). While missense changes seem to be more frequent in severe SIOD cases, frameshift and truncation mutations predominate in patients with mild phenotypes (7).

SIOD patients suffer from severe renal pathology (8) and eventually defective immunity that may cause recurrent infections (9). Although lymphopenia is a frequent finding in SIOD patients (10), the mechanism remains unknown, but some authors suggest that it may be due to either T-cell intrinsic or hematopoietic stem cell defects (11). Before kidney transplantation, patients receive immunosuppressive therapy, which can delay, but not fully prevent, kidney failure (12), probably due to the important roles of SMARCAL1 in kidney development (8). Under these circumstances, it is not easy to determine if lymphopenia is T-cell-intrinsic or secondary to hematopoietic stem cell defects or immunosuppression, and whether the latter should be maintained after kidney transplantation, considering the risk of associated life-threatening infectious events. While SMARCAL1 biology has been addressed in renal tissue pathology because of its relevance to the main life-threatening symptoms (8), it has not been studied deeply in T lymphocytes, because defective immunity is not present in all patients. Low responses to T-cell mitogens and defective IL-7 receptor expression and thus signaling have been reported in T cells from SIOD patients (13), but impaired T-cell development could explain such findings. Early membrane-proximal signaling has not been specifically studied in SIOD patients, likely because SMARCAL1 is a nuclear DNA helicase, so mechanistically it is unlikely to be involved in cytoplasmic events. Deficiencies of enzymes that participate in DNA damage and repair often cause T- and B-cell lymphopenia and thus opportunistic infections (14), but TCR signaling events remain unaffected (15).

Here we report a new SIOD patient with a homozygous c1920_1921insG frameshift mutation in *SMARCAL1* that generates a stop codon and likely leads to the synthesis of a truncated protein without the helicase domain. The patient received immunosuppressive treatment due to kidney transplantation and showed lymphopenia. We generated an immortalized T-cell line that allowed us to study if T-cell signaling defects in SIOD were T-cell-intrinsic or -extrinsic (i.e., induced by immunosuppression or lymphopenia). The results indicated that T-cell receptor (TCR)-mediated signaling was normal in SIOD-derived immortalized T cells, suggesting that the T-cell immunodeficiency is not due to intrinsic early TCR signaling defects, but rather to extrinsic defects caused by immunosuppression or lymphopenia.

## Materials and methods

### Case report

**Figure 1:**
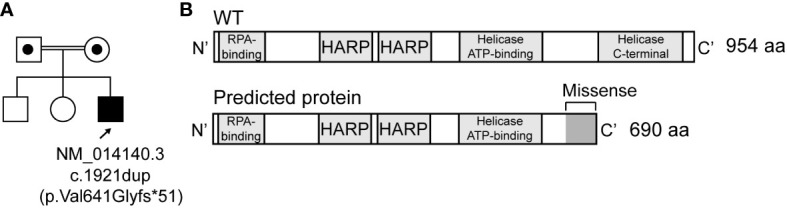
Homozygous mutations most likely cause a frameshift in *SMARCAL1*. **(A)** Pedigree for studied individuals, including the germline mutation (NM_014140.3, c.1921dup). Circles and squares indicate females and males, respectively. **(B)** Wild type and predicted SMARCAL1 proteins.

The patient was the third child of healthy consanguineous parents, with two healthy untested siblings. No prenatal pathology was detected during pregnancy (APGAR test 9/10). Born by urgent cesarean section due to oligohydramnios at week 33 of gestation with low weight (1,200 g; height, 38 cm). He was admitted to Neonatology for 30 days due to an intrauterine growth delay. At 3 years of age, he was diagnosed with spondyloepiphyseal dysplasia and short stature. Also, subclinical hypothyroidism and empty sella syndrome with growth hormone deficit and a short lower segment. At 5 years of age, he presented with cortico-resistant nephrotic syndrome with rapid evolution to secondary chronic kidney disease with hypertension that progressed to kidney failure at 8 years of age. The renal biopsy determined focal segmental glomerulosclerosis. Genetic testing identified a homozygous mutation (c.1921dup) in *SMARCAL1*, a diagnostic of SIOD (**Figure 1A**). This mutation was previously reported in a compound heterozygote Spanish SIOD patient (16). The insertion causes a frameshift and a missense sequence of around 50 amino acids within the region that codifies for the Helicase ATP-dependent region (Mutation Taster prediction), and the loss of the Helicase C-terminal region (**Figure 1B**).

He received a cadaveric donor kidney transplantation at the age of 9 and responded well to low immunosuppressants (1.4 mg/kg prednisone, 0.4 mg/kg tacrolimus, and everolimus 2–3 ng/ml) and other symptomatic treatments. Three months post-transplantation he presented with normal graft function, serum creatinine of 0.37 mg/dl, and asymptomatic bacteriuria in relation to the JJ catheter in ureteral implant, uncomplicated non-specific pneumonia, and transitory BK viruria. He did not have opportunistic infections and his HLA antibodies were negative. He only had a partial focal seizure at 9 years old that was resolved with treatment 18 months post-transplantation. A basic immunological test determined an inverted CD4/CD8 ratio, CD3 and CD19 lymphopenia, decreased proliferation of T lymphocytes, and decreased oxidation capacity of neutrophils. At age 11, he was admitted due to an acute respiratory process with hypoxemia. A lung CT scan showed normal perfusion ventilation. He maintained clinical hypoxemia. Secondarily, he developed moderate pulmonary hypertension, left ventricular hypertrophy, and mild ventricular dysfunction. Two months later, he was readmitted due to severe neutropenia, so everolimus was suspended. The second lung CT scan showed a diffuse ground glass pattern compatible with cellular bronchiolitis. Laboratory tests for *Aspergillus*, pneumocystis, respiratory viruses, and mycoplasma were negative. He died at 13 years of age due to a cardiorespiratory arrest with a functioning graft.

### PBMC isolation and immunophenotyping

Peripheral blood mononuclear cells (PBMC) were isolated by centrifugation on a Ficoll-Hypaque (GE Healthcare, Little Chalfont, United Kingdom) gradient. Blood samples were obtained after written informed consent from all participants or their tutors, according to the local ethics policy guidelines and the Declaration of Helsinki. Multiparametric flow cytometry was performed with mAbs against CD3 (UCHT-1), CD4 (13B8.2), CD19 (J3-119), CD45RA (ALB11), CD45RO (UCHL-1) and CD16 (3G8) from Beckman Coulter (Brea, Calif, USA); αβTCR (IP26) from Invitrogen/Thermo Fischer Scientific (Waltham, Mass, USA); γδTCR (11F2), IgD (IA6-2), CD31 (WM59), CD27 (M-T271), CD56 (B159), and CD8 (RPA-T8) from BD Biosciences (San Jose, Calif). Data were acquired with a FACSCalibur flow cytometer (BD Biosciences) at the Center for Cytometry and Fluorescence Microscopy of Complutense University of Madrid (Spain) and analyzed with FlowJo software (TreeStar, Ashland, Ore, USA).

### Generation of immortalized T-cell lines

Human T-lymphotropic virus type 1 (HTLV-I) cell lines were generated as described (17). Briefly, PBMC were stimulated with 10 μg/ml phytohemagglutinin-L (PHA-L) from *Phaseolus vulgaris* (only at day 0) from Sigma-Aldrich (St. Louis, Mo) for 24 h and co-cultured with gamma-irradiated (150 Gy) HTLV-1-producing cell line (MT2) at 1:2 ratio in RPMI 1640 medium from Lonza (Basel, Switzerland), supplemented with 100 IU/ml recombinant interleukin-2 (rIL-2) (provided by Craig W. Reynolds, Frederick Cancer Research and Development Center, NCI, NIH, Frederick, Maryland, USA), 10% fetal bovine serum (FBS) and 1% L-glutamine and antibiotic–antimycotic from Life Technologies (Carlsbad, Calif, USA). Cells were maintained at 1–2 × 10^6^ cells/ml and culture medium supplemented with 100 IU/ml rhIL-2 was refreshed twice per week.

### Genomics

Genomic DNA for mutation determination was extracted from 3 million immortalized T cells using QIAamp^®^ DNA Mini (Qiagen, Düsseldorf, Germany) and resuspended in H_2_O. A total of 100 ng were used for PCR amplification using Easy™ Oligo primers purchased from Sigma Aldrich (Burlington, MA, USA), designed as indicated in **Supplementary Table S1**, and TAQ PCR MASTER KIT (1,000, Cat. No. 201443) from Qiagen. PCR products were Sanger sequenced in the Genomics Unit of the Complutense University of Madrid (Spain) on a 3730xl DNA Analyzer and studied by Chromas software.

To analyze *SMARCAL1* expression, total RNA was isolated using the RNeasy Mini Kit (250, Cat. No. 7410) from Qiagen and resuspended in H_2_O. A total of 2 µg was used for reverse transcription using the High-Capacity cDNA Reverse Transcription Kit from Applied Biosystems™ (Waltham, MA, USA). Different amounts of cDNA were used for PCR amplifications using Easy™ Oligo primers purchased from Sigma Aldrich (**Supplementary Table S1**) and TAQ PCR MASTER KIT (1,000, Cat. No. 201443) from Qiagen. PCR products were loaded onto a 2% agarose (Tris-acetate-EDTA buffer) gel with a 1/30,000 dilution of SYBR^®^ Green nucleic acid gel stain from Invitrogen (Waltham, MA, USA) and visualized with the GelDoc Go Gel Imaging System from BIO-RAD (Hercules, CA, USA).

### T-cell function assays

TCR signaling was studied using standard protocols (18). Briefly, 0.2 × 10^6^ PBMC or immortalized T cells were plated in RPMI-1640 supplemented with 10% FBS in flat-bottom 96-well plates coated with 10 μg/ml anti-CD3 mAb (UCHT-1 from BD Biosciences) and stimulated for 24 h. T-cell activation was analyzed by flow cytometry with anti-CD69 mouse anti-human antibody (L-78 from BD Biosciences). Alternatively, 0.2 × 10^6^ PBMC were plated in RPMI-1640 supplemented with 10% FBS in flat-bottom 96-well plates coated with 1 μg/ml anti-CD3 mAb (OKT-3; eBioscience, Waltham, Mass, USA), or with 5 μg/ml PHA-L, or with 10 ng/ml phorbol 12-myristate 13-acetate (PMA) and 1 mM ionomycin or 10 ng/ml superantigens (*Staphylococcal* Enterotoxin B or E; Toxin Technology, Inc, Sarasota, Fla) and stimulated for 5 days, and the percentage of blastic cells within the lymphocyte SSC-H *vs* FSC-H gate was quantified by flow cytometry.

### DNA repair assay

Immortalized T-cell lines were harvested in an RPMI-1640 medium and treated with 10 Gy gamma irradiation (using the Irradiator Gammacell 1000 of the Central Radioactive Facility of Complutense University of Madrid, Spain) as described (19). DNA repair was evaluated at different time points, the phosphorylation of γH2AX was evaluated by intracellular flow cytometry using Alexa Fluor^®^ 647 Mouse anti-H2AX antibody (pS139 clone, BD Biosciences) and eBioscience™ Foxp3/Transcription Factor Staining Buffer Set and protocol (BD Biosciences). Data were acquired with a FACSCalibur flow cytometer and analyzed with FlowJo software.

Standard biosecurity and institutional safety procedures have been adhered to, following European and Universidad Complutense guidelines.

## Results

### Primary T cells

The patient showed selective severe T-cell lymphopenia at 9 years of age (**Table 1**).

**Table 1:** Immunological phenotype.

Lymphocytes [cells/mm^3^ (%)]	Patient (9 y)	Normal range (5-10 y)
	1,030	1,200–4,700
T (CD3^+^)	**235 (22.8)**	800–4,000 (55–97)
CD4^+^	**119** (51)	400–2,500 (26–61)
CD8^+^	**94** (40)	200–1,700 (13–47)
TCRαβ^+^	**223** (**95**)	600–3,700 (44–92)
TCRγδ^+^	**9** (4)	27–960 (2–24)
CD4/CD8 ratio	1.3	1.2–2.7
B (CD19^+^)	**525** (**51**)	228–516 (10–18)
CD27^−^ IgD^+^	378 (72)	133–389 (69–80)
CD27^+^ IgD^+^	**63** (12)	22–43 (7.5–12)
CD27^+^ IgD^−^	**47** (9)	16–31 (5.2–12)
CD27^−^ IgD^−^	**37** (**7**)	10–24 (3.5–6.6)
NK (CD56^+^ CD3^neg^)	**62** (6)	70–590 (2–31)
CD56^+^ CD16^neg^	8 (**13**)	7–68 (9.9–11.6)
CD56^+^ CD16^+^	**47** (**76**)	62–537 (89–91)

Bold = out of range. Normal ranges from Morbach et al., (20) Schatorje et al., (21), and Tosato et al. (22).

**Figure 2:**
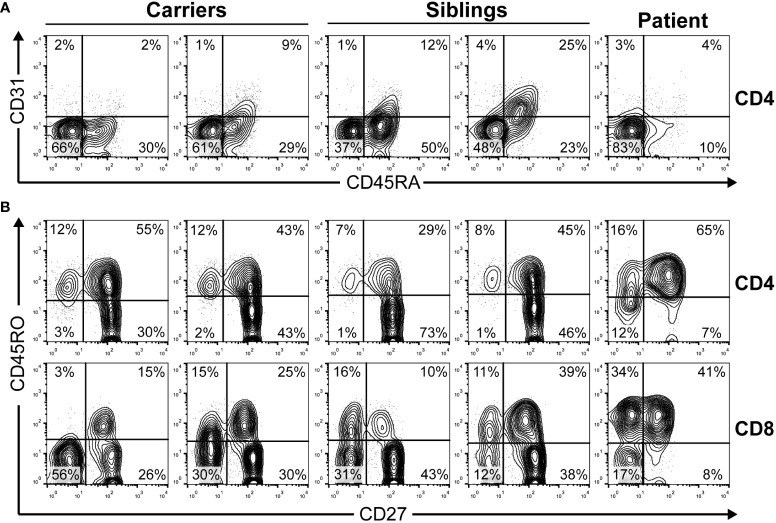
The peripheral blood phenotype of T cells was determined by extracellular flow cytometry in the indicated family members. **(A)** Recent thymic emigrants CD4 T cells defined as CD45RA+CD31+ lymphocytes as reported (23). **(B)** T-cell maturation stages defined in CD4 and CD8 bright T-cells as naive (CD45RO−CD27+), central memory (CD45RO+CD27+), effector memory (CD45RO+CD27−), or effector (CD45RO−CD27−). Carriers, 39–40 years old (yo); siblings, 12–15 yo; patient, 9 yo.

Accordingly, thymus output measured as recent thymic emigrants was strongly reduced and most peripheral T cells were CD45RO+ (memory) in the patient (**Figure 2**). The evolution of the leucocyte and lymphocyte cell numbers indicated lymphopenia and a decrease after immunosuppression (see **Figure S1** in **Supplementary Material**).

### Immortalized T cells

**Figure 3:**
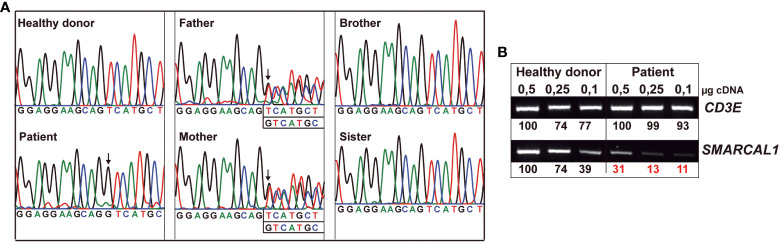
Heterozygous and homozygous *SMARCAL1* c.1921dup mutations in immortalized T cells. **(A)** Genomic DNA *SMARCAL1* Sanger sequencing of immortalized T-cell lines from the indicated donors. **(B)** Different amounts of T-cell line cDNA from healthy donors and patients were amplified by PCR using primers for *CD3E* as an internal control and *SMARCAL1*, as indicated in **Table S1** (see **Supplementary Material**). The numbers represent the intensities of *SMARCAL1 vs CD3E* PCR product bands.

To understand the relevance of SMARCAL1 in TCR signaling *ex vivo*, excluding immunosuppression effects, HTLV-I-immortalized T-cell lines were generated from all family members and an unrelated healthy donor. All cell lines showed similar cell growth kinetics (see **Figure S2** in **Supplementary Material**). *SMARCAL1* Sanger sequencing of all immortalized T-cell lines confirmed the expected segregation of the mutation (compare **Figure 1A** and **Figure 3A**). To estimate the effect of the homozygous mutation on *SMARCAL1* transcription, the sequence containing the mutation was amplified by PCR using different amounts of cDNA from immortalized, healthy, and patient T cells. *CD3E* was used as a T cell-specific internal positive control. The results showed a strong reduction of *SMARCAL1* PCR product in the patient compared to a healthy donor, relative to *CD3E* (**Figure 3B**).

**Figure 4:**
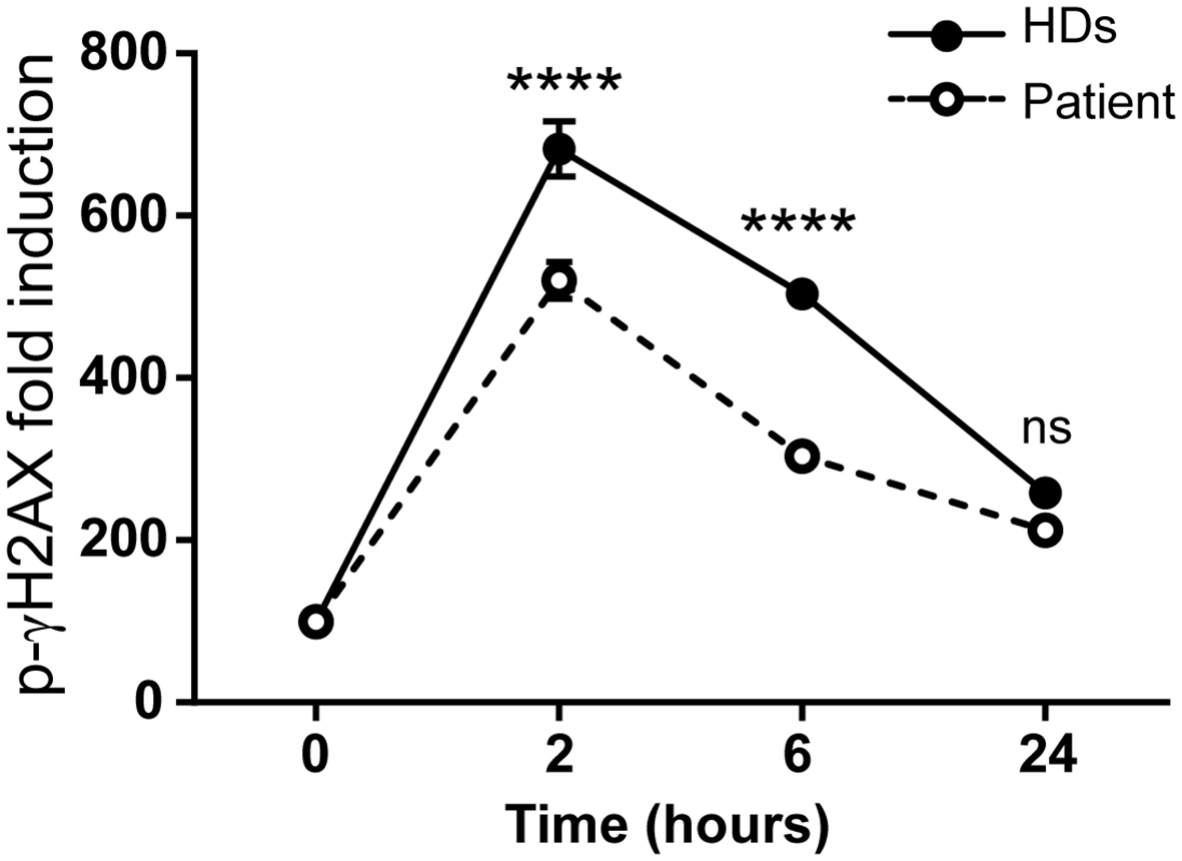
The *SMARCAL1* c.1921dup mutation affects gamma irradiation DNA repair in T-cell lines. Immortalized T-cell line DNA repair after gamma irradiation treatment was evaluated by intracellular p-γH2AX levels by flow cytometry at different time points and the geometric mean fluorescence intensity (geo-MFI) relative to non-treated cells was calculated. HDs = 3; n = 6; ****p<0.0001; ns, not significant.

DNA repair kinetics can be studied by treating cells with insults that induce DNA breaks and recruit the phosphorylated histone γH2AX (p-γH2AX) (24). As SMARCAL1 is a component of the DNA damage response, epithelial SMARCAL1-deficient cells are hypersensitive to replication stress agents like hydroxyurea (HU) (25, 26), and increased DNA damage markers but not survival have been described in *Smarcal1* KO mouse thymocytes after gamma radiation (11). Therefore, we studied DNA damage response sensitivity *in vitro* in the immortalized T-cell lines. To that end, cells were treated with 10 Gy gamma irradiation and p-γH2AX levels were measured at different time points. At early time points, gamma irradiation induced significantly lower p-γH2AX levels in immortalized patient T cells compared to healthy donors (**Figure 4**).

Taken together, these results validated the immortalized T-cell lines for the *SMARCAL1* mutation, gene expression, and functional defects.

### Primary *vs* immortalized T cells

Next, TCR function was studied in primary and immortalized T cells. Early (CD69 induction) and late (proliferation against different stimuli) events were studied after TCR-dependent (anti-CD3, PHA) or independent (PMA and ionomycin or ION) stimuli. CD69 induction (**Figure 4**, top) and T-cell proliferation (see **Figure S3** in **Supplementary Material**) after anti-CD3 or PHA stimulation were strongly impaired in primary T cells from the patient compared to carriers and siblings. The defect was TCR-dependent, as PMA+ION induced normal responses in the primary T cells of the patient. In sharp contrast, immortalized T cells analyzed in parallel for CD69 induction showed completely normal responses to TCR engagement (**Figure 4**, bottom). These results indicate that SMARCAL1 does not have an intrinsic role in TCR signaling.

## Discussion

**Figure 5:**
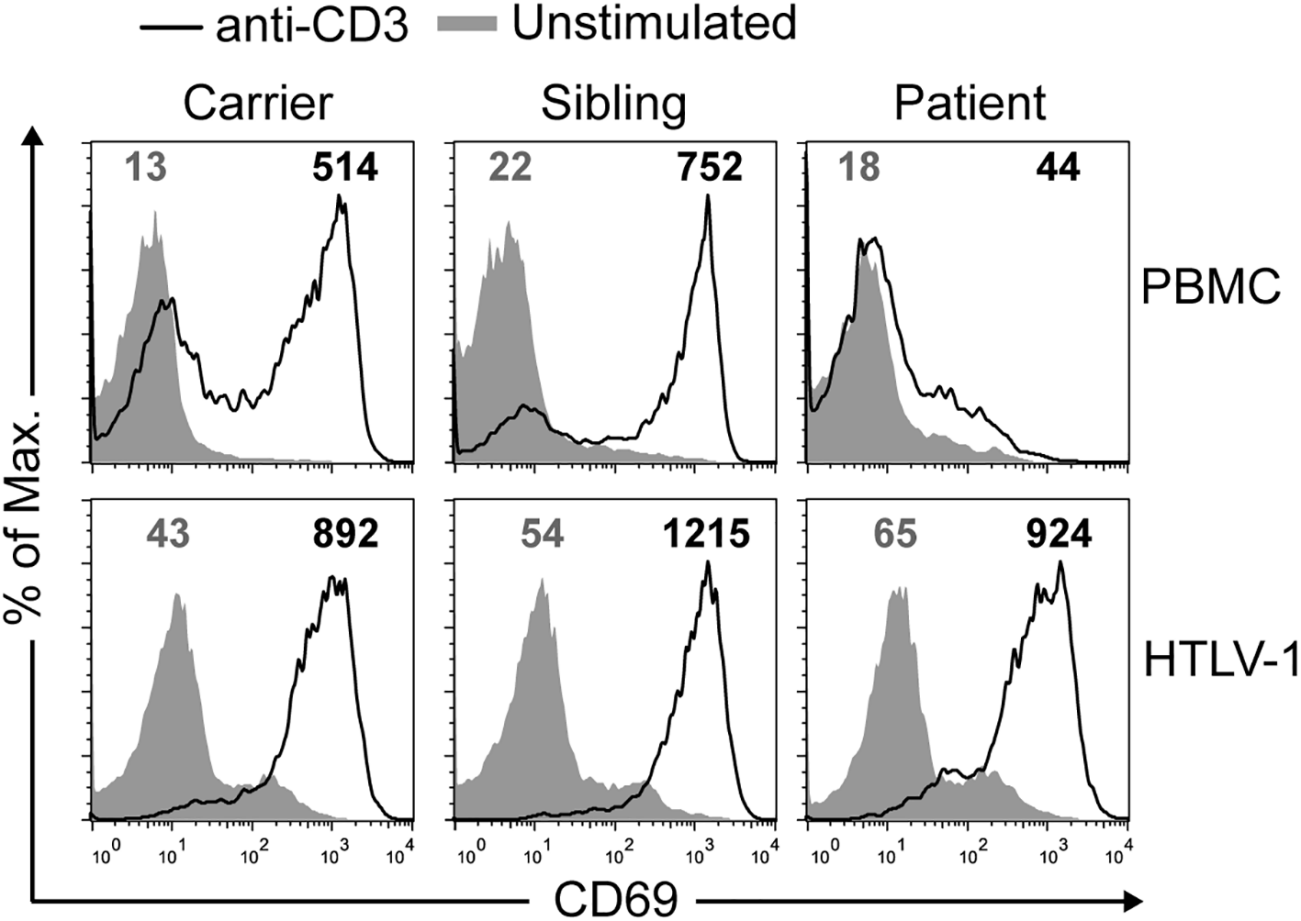
Normal signaling in immortalized but not primary SIOD T cells. CD69 mean fluorescence intensity (MFI) was quantified in non-stimulated cells (gray lines) and after stimulation (black lines) with anti-CD3 antibody for 24 h by flow cytometry on primary (top) or immortalized (bottom) T cells. This experiment was repeated twice with similar results.

We have shown that a lethal homozygous *SMARCAL1* mutation causing severe kidney disease and lymphopenia in a boy did not impair TCR signaling in a T-cell model derived from the patient. The immortalized T cells were shown to carry the same mutations originally detected in the patient (**Figure 3**), which impaired *SMARCAL1* gene expression (**Figure 3B**) as well as the response of the cells to gamma irradiation (**Figure 4**), but not TCR-mediated signaling (**Figure 5**). Impaired response to gamma-irradiation has also been reported in other cell systems (shRNA and knockdown chicken cell lines), where a reduction or loss of SMARCAL1 increased radiosensitivity (27, 28).

Similar results were found with immortalized T cells derived from ataxia telangiectasia patients (15). Despite a long-term belief in the field that the affected molecule (ATM, a nuclear kinase activated by double-strand breaks or oxidative stress, which phosphorylates several targets including SMARCAL1) was involved in TCR or BCR signaling and thus caused lymphopenia and immunodeficiency, we showed that immortalized T cells derived from patients carried the causing mutations and thus preserved the sensitivity to ionizing radiation of ataxia telangiectasia cells, but did not show any intrinsic immune functional defects when stimulated through their TCR.

As TCR-mediated signaling is SMARCAL1-independent, how can the observed immune defects observed in SIOD patients be connected to SMARCAL1 function? This includes frequent lymphopenia and less frequent immunodeficiency (i.e., susceptibility to infection; see **Table 2**). Our hypothesis is that SMARCAL1 impairs nuclear functions required for early T-cell development checkpoints that require strong cellular expansion. Such a developmental defect would cause lymphopenia and, in some cases, also immunodeficiency. This hypothesis has been proved right by others for a different DNA helicase termed BLM involved in Bloom’s syndrome in humans, which is associated with mild lymphopenia and an increased number of infections (54). T-lineage-specific mouse BLM ablation was shown to decrease thymocyte numbers due to a developmental block at the T-cell β-selection checkpoint (55). The hypothesis has not been directly addressed in SIOD patients, but some results in *Smarcal1*-deficient mice strongly point to a hematopoietic progenitor cell defect (11). Our hypothesis could be tested by studying SIOD T-cell differentiation using CD34+ lymphoid precursors in artificial thymic organoids as reported elsewhere for intrinsic (such as reticular dysgenesis) *vs* extrinsic (such as DiGeorge syndrome) T-cell lymphopenia (56). Experiments are underway to test this hypothesis. The fact that early thymic emigrants in peripheral blood are essentially absent in our patient (**Figure 2A**) and other studied patients (13) lends support to the hypothesis of an SMARCAL1-dependent expansion defect in T-cell development.

**Table 2:** Immunological features of SIOD patients.

	No. of patients	Lymphopenia	Recurrent
			infections
New SIOD patient	1	1/1	1/1
Castellano-Martinez et al. (29)	2	0/2	0/2
Malhotra et al. (30)	1	1/1	0/1
Hara-Isono et al. (31)	2	1/2	1/2
Wang et al. (32)	1	1/1	0/1
Ramdeny et al. (33)	1	1/1	0/1
Bertulli et al. (34)	2	2/2	2/2
Prato et al. (35)	2	2/2	0/2
Xiong et al. (36)	1	1/1	1/1
Jin et al. (6)	1	1/1	0/1
Haffner et al. (37)	1	0/1	0/1
Arad and Pirzadeh (38)	1	0/1	0/1
Power et al. (10)	2	2/2	1/2
Lipska-Zietkiewicz et al. (9)	34	25/30	17/31
Liu et al. (39)	1	1/1	1/1
Barraza-Garcia et al. (40)	1	1/1	1/1
Carroll et al. (41)	1	1/1	1/1
Pedrosa et al. (42)	1	0/1	1/1
Santangelo et al. (43)	1	1/1	0/1
Baradaran-Heravi et al. (44)	5	4/5	3/5
Yue et al. (45)	1	1/1	1/1
Lev et al. (46)	3	3/3	1/3
Lucke et al. (47)	3	3/3	1/3
Basiratnia and Fallahzadeh (48)	1	2/2	0/2
Clewing et al. (49)	20	16/20	8/20
Petty et al. (50)	1	1/1	1/1
Ehrich et al. (51)	2	2/2	2/2
Spranger et al. (52)	5	2/5	2/5
Schimke et al. (53)	1	1/1	1/1
**Total**	**99**	**77/99 (78%)**	**47/99 (47%)**

It may be argued that our findings refer to a single patient, but numerous single patient studies have revealed crucial pathways underlying physiological and pathological processes by establishing a causal relationship between the candidate genotype and the clinical phenotype *via* a relevant cellular phenotype, as we show here (57). Also, the literature has not addressed the role of SMARCAL1 in TCR-mediated signaling in patient-derived immortalized T cells, as we have done here for the first time. An obvious caveat to using a single patient is, naturally, that different mutations may differently affect T-cell development since disease severity has been shown to be inversely proportionate to overall SMARCAL1 activity (7). Thus, the spectrum of the disease may vary from mild phenotypes, as observed in patients with frameshift and truncation mutations (43), to very severe phenotypes in patients with homozygous missense mutations, as described in our work and others' (7, 58).

Although HTLV-1 transformation could affect T-cell function by itself, the use of HTLV-1 T-cell lines as *in vitro* models of human T lymphocytes has been validated in the past (59) and we have extensively demonstrated that the immortalization process does not affect the functional status of proximal TCR (15, 60–65) and other (18, 66) signaling pathways. We do not rule out that SMARCAL1 has a role in T-cell proliferation, however, and this would fit better with the reported role for that nuclear protein, although we favour the hypothesis of an SMARCAL1-dependent expansion defect in early T-cell development.

The immortalized T cells carrying the lethal *SMARCAL1* mutation characterized here may help in understanding the distinct biological role of SMARCAL1 in different cell types and developing rational therapies for the T-cell immunological dysfunction of SIOD patients with severe mutations. However, **Table 2** clearly shows that there is a great variability in terms of T-cell lymphopenia, which is present in most SIOD patients (78%), and T-cell immunodeficiency as estimated by susceptibility to infections (present only in 47% of the patients). Thus, immunosuppression regimens must necessarily be personalized according to the immune profile of the patient.

## Conclusions

T-cell receptor early signaling in Schimke immuno-osseous dysplasia is SMARCAL1-independent, as demonstrated by the fact that transformed T cells carrying lethal *SMARCAL1* mutations showed impaired *SMARCAL1* gene expression and impaired response to gamma irradiation but not impaired T-cell receptor signaling for CD69 induction.

## Data availability statement

The raw data supporting the conclusions of this article will be made available by the authors, without undue reservation.

## Ethics statement

This study was reviewed and approved by CEIm Hospital Clínico San Carlos. Written informed consent to participate in this study was provided by the participants’ legal guardian/next of kin.

## Author contributions

JRR and ER-O conceived the experimental study. AVM, AJ-R, and MSM designed the experiments and analyzed the data. JRR and AVM wrote the manuscript. All authors contributed to the article and approved the submitted version.

## Funding

This work was supported by grants from the Ministerio de Economía y Competitividad (MINECO PID2021-125501OB-I00 and RTI2018-095673-B-I00), the Comunidad Autónoma de Madrid (CAM B2017/BMD3673), and the Asociación Española Contra el Cáncer (AECC PROYE20084REGU). AVM was supported by the Complutense/Harvard University (CT46/15); AJ-R by the MINECO (grant no. BES-2012-055054).

## Acknowledgments

We thank Alejandro Briones for manuscript revision, Carmen Alonso and Julia Fernandez-Boraita for technical help, and the patient, his family, the Asociación Española de Displasias Óseas Minoritarias (www.facebook.com/asociacionaedom/), and clinicians for their collaboration.

## Conflict of interest

The authors declare that the research was conducted in the absence of any commercial or financial relationships that could be construed as a potential conflict of interest.

## Publisher’s note

All claims expressed in this article are solely those of the authors and do not necessarily represent those of their affiliated organizations, or those of the publisher, the editors and the reviewers. Any product that may be evaluated in this article, or claim that may be made by its manufacturer, is not guaranteed or endorsed by the publisher.
