# Supplementary material for: T-cell receptor signaling in Schimke immuno-osseous dysplasia is SMARCAL1-independent
Source: Front Immunol. 2022 Oct 18;13:979722. doi: 10.3389/fimmu.2022.979722 (PMC9623027; doi:10.3389/fimmu.2022.979722)
Supplement: Supplementary file 1 [file DataSheet_1.docx]

Supplementary Figures and Tables

**
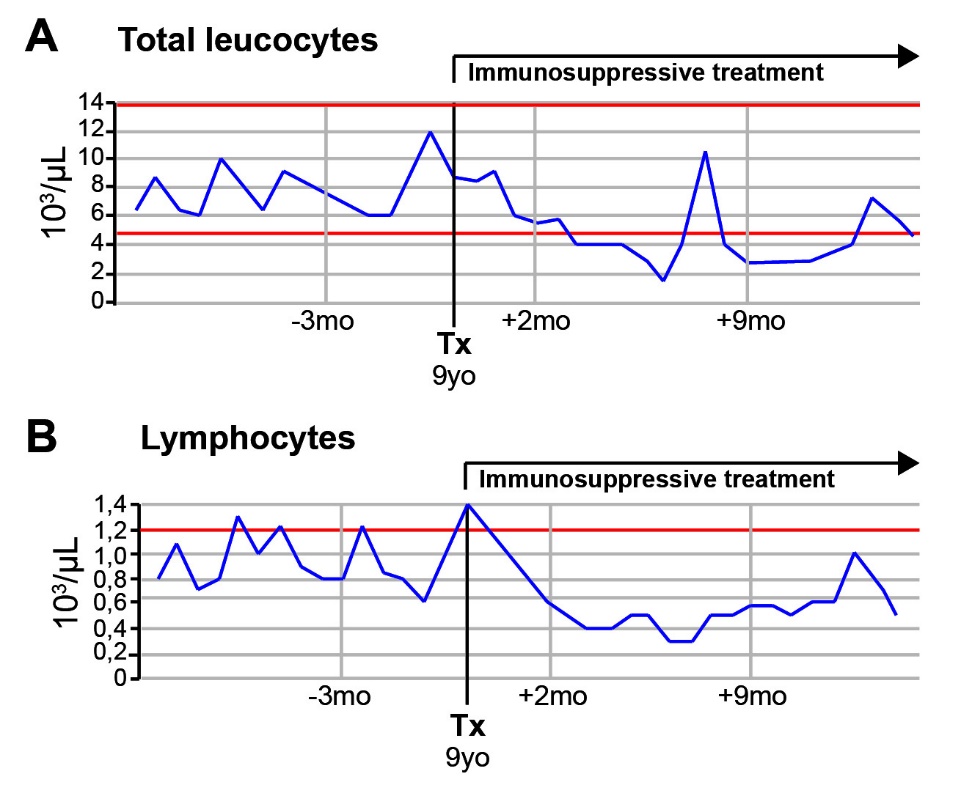
**

**Supplementary Figure S1.** Evolution of immune cell number before and after transplant (Tx) and immunosuppression treatment. **(A)** Number of total leucocytes. **(B)** Number of lymphocytes. Red lines indicate normal values for his age range. mo: months; yo: years-old.


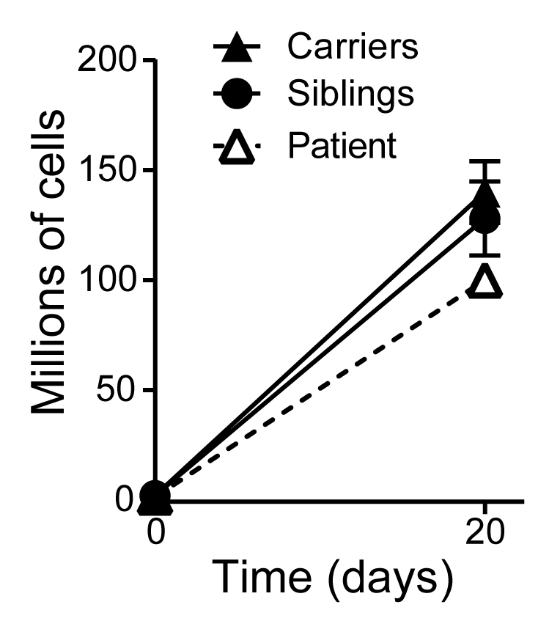


**Supplementary Figure 2.** Efficiency of HTLV-1 transformation. HTVL-1 immortalized T cells were harvested and counted using trypan blue to identify dead cells from carriers (n=2), siblings (n=2) or patient (black triangle, black circle, or white triangle, respectively) and did not differ significantly from each other.

**
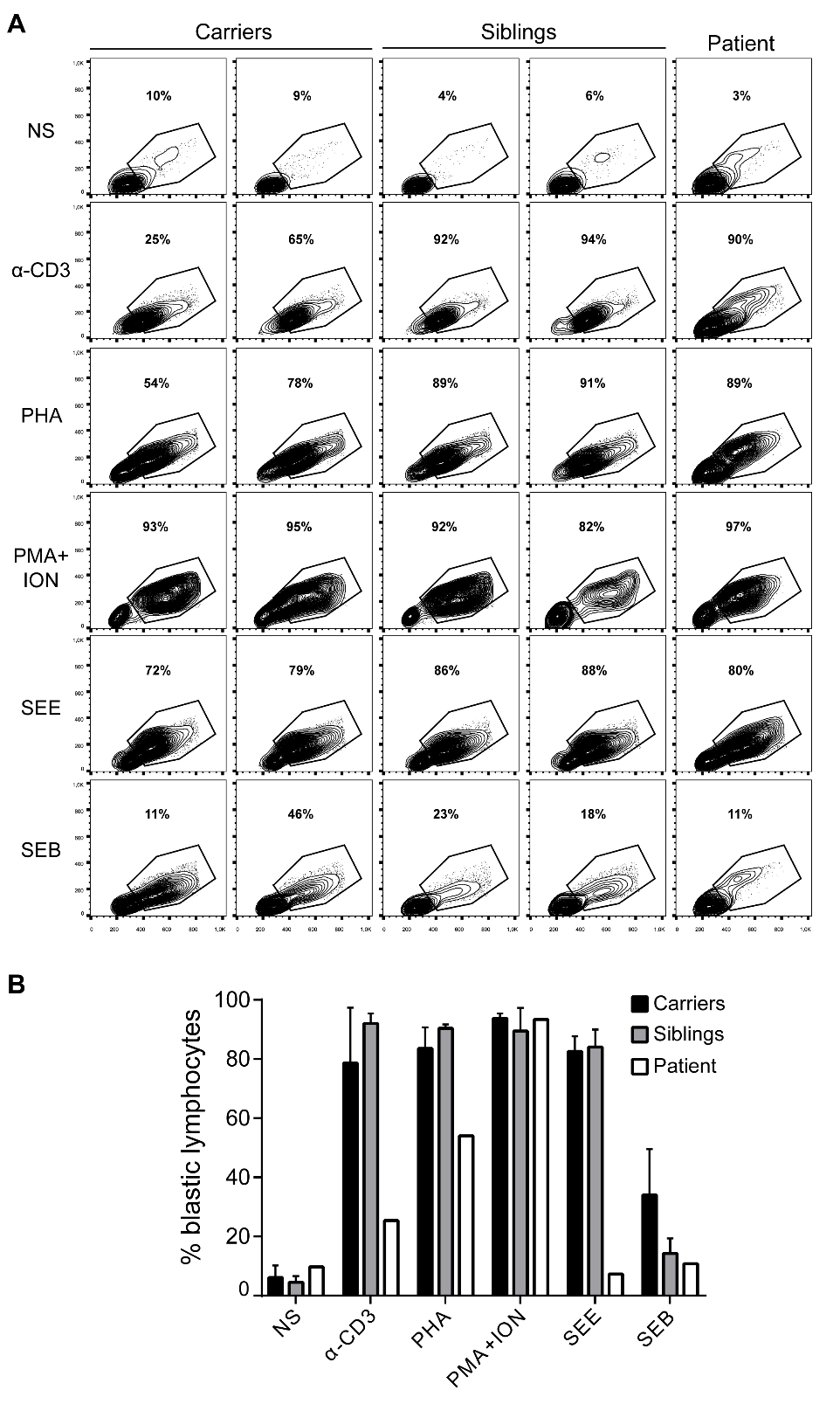
**

**Supplementary Figure S3.** Lymphocyte proliferation in response to mitogens. Carriers (n=2), siblings (n=2) and patient PBMC were cultured in RPMI-1640 supplemented with 10% FBS for 5 days after stimulation with 1 µg/mL plate-coated anti-CD3 (OKT3), 5 µg/mL *Phaseolus vulgaris* leucoagglutinin (PHA), 10 ng/mL phorbol 12-myristate 13-acetate (PMA) and 1 mM ionomycin or 10 ng/mL *Staphylococcal* Enterotoxin B or E superantigens (SEB or SEE, respectively). % of blastic cells within the lymphocyte gate **(A)** was quantified by flow cytometry **(B)**.

| **TABLE S1.** Primers | | | |  |  |  |  |
| --- | --- | --- | --- | --- | --- | --- | --- |
|  | |  | | | | | |
| Material | Target | | Direction | Primer sequence (sequence 5'->3') | T_M_ (ºC) | T_A_ (ºC) for PCR | PCR product size (bp) |
| gDNA | *SMARCAL1* | | Forward | GTTGGACTGGGCCTGAAAGG | 68,0 | 66 | 574 |
|  |  |  | Reverse | CCACATTCTTTGAGGGATTCGGA | 69,9 |  |  |
| cDNA | *SMARCAL1* | | Forward | CGCAGAGCTCTACACGCAG | 64,4 | 62 | 338 |
|  |  |  | Reverse | GAATGAGGGCATCTTTCTGCT | 65,2 |  |  |
| cDNA | *CD3E* | | Forward | TCGGTGGCCACAATTGTCAT | 68,6 | 64 | 358 |
|  |  |  | Reverse | CCATGAGGCTGAGGAACGAT | 66,4 |  |  |
| gDNA or cDNA refers to genomic or complementary DNA, respectively. T_M_: melting temperature; T_A_: annealing temperature. | | | | | | | |
